# Targeting TBP-Associated Factors in Ovarian Cancer

**DOI:** 10.3389/fonc.2014.00045

**Published:** 2014-03-11

**Authors:** Jennifer R. Ribeiro, Lindsay A. Lovasco, Barbara C. Vanderhyden, Richard N. Freiman

**Affiliations:** ^1^Pathobiology Graduate Program, Brown University, Providence, RI, USA; ^2^Molecular and Cellular Biology and Biochemistry, Brown University, Providence, RI, USA; ^3^Cellular and Molecular Medicine, University of Ottawa, Ottawa, ON, Canada; ^4^Centre for Cancer Therapeutics, Ottawa Hospital Research Institute, Ottawa, ON, Canada

**Keywords:** TAF2, TAF4, TAF4B, TAF9, TBP-associated factors, TFIID, differentiation, ovarian cancer

## Abstract

As ovarian tumors progress, they undergo a process of dedifferentiation, allowing adaptive changes in growth and morphology that promote metastasis and chemoresistance. Herein, we outline a hypothesis that TATA-box binding protein associated factors (TAFs), which compose the RNA Polymerase II initiation factor, TFIID, contribute to regulation of dedifferentiation states in ovarian cancer. Numerous studies demonstrate that TAFs regulate differentiation and proliferation states; their expression is typically high in pluripotent cells and reduced upon differentiation. Strikingly, TAF2 exhibits copy number increases or mRNA overexpression in 73% of high-grade serous ovarian cancers (HGSC). At the biochemical level, TAF2 directs TFIID to TATA-less promoters by contact with an Initiator element, which may lead to the deregulation of the transcriptional output of these tumor cells. TAF4, which is altered in 66% of HGSC, is crucial for the stability of the TFIID complex and helps drive dedifferentiation of mouse embryonic fibroblasts to induced pluripotent stem cells. Its ovary-enriched paralog, TAF4B, is altered in 26% of HGSC. Here, we show that *TAF4B* mRNA correlates with Cyclin D2 mRNA expression in human granulosa cell tumors. TAF4B may also contribute to regulation of tumor microenvironment due to its estrogen-responsiveness and ability to act as a cofactor for NFκB. Conversely, TAF9, a cofactor for p53 in regulating apoptosis, may act as a tumor suppressor in ovarian cancer, since it is downregulated or deleted in 98% of HGSC. We conclude that a greater understanding of mechanisms of transcriptional regulation that execute signals from oncogenic signaling cascades is needed in order to expand our understanding of the etiology and progression of ovarian cancer, and most importantly to identify novel targets for therapeutic intervention.

## Introduction

Ovarian cancer is the most deadly reproductive cancer. Although progress has been made in understanding its etiology and progression, there has been no improvement in patient overall survival since the implementation of taxane–platinum therapy in the 1990s (1). For this reason, novel approaches are required to make headway in this challenging disease. Many researchers are taking advantage of the genomic data in the cBioPortal for Cancer Genomics assembled by The Memorial Sloan Kettering Cancer Center (MSKCC) to identify potential new targets for treatment, which has greatly contributed to our understanding of the complex genetic mechanisms governing ovarian cancer (2, 3). However, targeting specific oncogenic pathways can be challenging, since resistance develops due to activation of compensatory pathways (4). These signaling pathways converge on transcriptional control of genes that regulate differentiation, proliferation, and apoptosis, as well as other cancer cell properties including migratory and invasive potential, immune response, angiogenesis, telomere maintenance, and energy metabolism. Although not without challenges itself, investigating cell-type specific mechanisms of global transcriptional regulation, which may potentially be disrupted to halt or reverse tumor progression, could open up a new field of investigation in ovarian cancer research.

**Figure 1:**
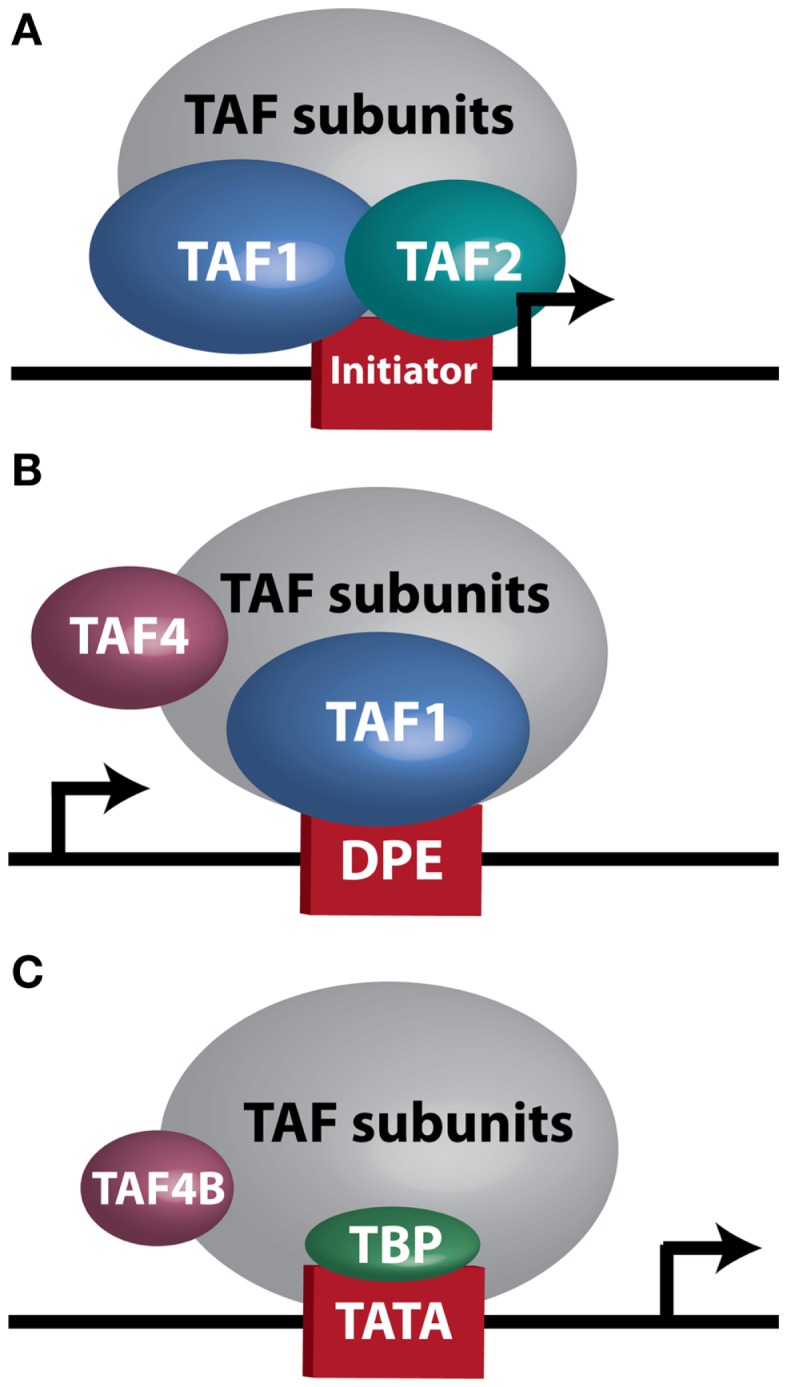
**TFIID sub-complexes**. Arrows indicate transcription start site. **(A)** A TAF1/TAF2 complex contacts Initiator at a TATA-less promoter. **(B)** TAF1 contacts a downstream promoter element (DPE) at a TATA-less promoter. TAF4 is also important for this interaction. **(C)** TAF4B substitutes for TAF4 in a TFIID complex bound at a TATA-box by TBP.

At the biochemical level, transcription is controlled by numerous core transcriptional complexes, such as TFIID, along with various cofactors. Studies in human cells and *Drosophila* initially revealed TFIID as an integral component of the core transcriptional machinery for RNA Polymerase II at mRNA encoding genes (5, 6), and demonstrated that it is composed of TATA-box binding protein (TBP) and multiple TBP-associated factors (TAFs) (7, 8). To date, 13–14 TAFs (9) and several tissue-specific variants (10) have been identified. *In vivo* genetic analyses unveiled a more complex role for TFIID in regulating tissue-specific and context-dependent transcriptional programs, demonstrating the existence of alternative TFIID complexes and tissue-specific TAFs (11–17). Three such complexes, which will be discussed in greater detail later in this manuscript, are illustrated in Figure 1. The realization that TFIID subunits regulate cellular processes in tissue-specific manners prompted research into TAF involvement in modulating tumor characteristics, including proliferation, differentiation, apoptosis, metastasis, and hormone response. The considerable variability seen in these reports, which are summarized in Table 1, further supports the notion that the plasticity of the TFIID complex allows for variation in transcriptional control depending on cellular context. However, perhaps due to this plasticity, our understanding of the contribution of the TFIID complex to tumorigenesis and cancer progression remains limited.

**Table 1:** **Summary of studies on TAFs in cancer**.

**TAF1**	
•	Frequent mutations in uterine serous carcinoma (18)
•	Compensatory for androgen withdrawal in prostate cancer/co-activator for androgen receptor (19)
•	Knockdown causes resistance to stress-induced apoptosis/reduces p27 (kip1) expression (20)
•	Interacts with HPV protein E2 (21, 22). Overexpression in cervical cancer cells modulates E2-dependent transcription (21)
•	Promotes MDM2 degradation of p53 (23). Promotes cell cycle progression by phosphorylating p53 and promoting its degradation (24)
•	Histone acetyltransferase (HAT) activity of TAF1 is important for cyclin D1 transcriptional activation and cell cycle progression (25)
•	TAF1 inactivation promotes DNA damage response and cell cycle arrest (26)
•	TAF1/TAF2 binds TATA-less SRC promoters that have Initiator elements. Transcription from SRC promoters is TAF1-dependent, and the HAT activity of TAF1 partly regulates transcription from SRC promoters (27)
•	Interacts with B-Myb and helps mediate activation of Myb-response genes, which regulate cell cycle (28)
•	Bound by c-Jun, which increases TFIID-driven transcription by de-repressing TAF1 repression of TBP binding to TATA-boxes (29)
•	Regulates transcription of cyclin A (30)
**TAF2**	
•	TAF1/TAF2 binds TATA-less SRC promoters with Initiator elements (27)
•	Yeast TAF2 required for transcription of B-type cyclins and cell cycle progression (31)
**TAF4 and TAF4B**	
•	TAF4 inactivation in adult mouse epidermis causes epidermal hyperplasia, upregulation of EGF family mitogens, malignant transformation of DMBA-induced papillomas, and appearance of invasive melanocytic tumors in DMBA-treated mice (32)
•	Estrogen upregulates TAF4B in mouse serous ovarian tumors (33)
•	TAF4B identified as a hub gene in head and neck squamous carcinoma associated with radiosensitivity (34)
•	TAF4B knockdown promotes migration of colon cancer cells *in vitro* by down regulation of the AP-1 target gene *ITG*α6 (35)
•	TAF4B is a c-Myc target gene in human glioblastoma cells and human promyelocytic leukemia cells (36)
**TAF6**	
•	72 kDa isoform causes growth suppression of normal and transformed breast epithelial cell lines due to novel interaction with the G2 arrest protein GADD45a (37)
•	72 kDa isoform forms a TFIID complex lacking TAF9; its elevated expression in Hela cells causes apoptosis, increased transcription of p21 and *GADD45*, and decreased *MDM2* transcription (38)
•	TAF6 and TAF9 necessary for transcriptional activation by p53 (39)
**TAF7 and TAF7L**	
•	TAF7 knockdown in androgen-independent prostate cancer cells reduces polyamine transport and causes resistance to methylglyoxalbisguanylhydrazone (MGBG)-induced apoptosis (40)
•	TAF7 is a co-activator for the mitogen C-JUN in HEK293 and COS cells (41)
•	TAF7L downregulated in 59% of male patients with acute myeloid leukemia (42)
**TAF9**	
•	Disruption of interactions between Hedgehog transcription factors (Gli proteins) and TAF9 reduces Gli/TAF9-dependent transcription, suppresses cancer cell proliferation, and reduces xenograft growth (43)
•	UV and IR disrupts hydrogen bonding between Thr18 and Asp21 on p53, reducing MDM2 binding to p53 and allowing recruitment of p53 co-activator, TAF9 (44)
•	TAF9 inhibits MDM2-mediated degradation of p53/acts as a co-activator of p53 (45)
•	72 kDa TAF6 isoform forms a TFIID complex lacking TAF9/its elevated expression in Hela cells causes apoptosis, increased transcription of p21 and *GADD45*, and decreased *MDM2* transcription (38)
•	TAF6 and TAF9 necessary for transcriptional activation by p53 (39)
•	TAF9 is a crucial co-activator for p53 (46)
**TAF10**	
•	Stimulates transcription from ERE-containing promoters (47)
**TAF12**	
•	Upregulated in colon cancer cell lines with RAS mutations or overexpression of mutant RAS; knockdown destabilizes TFIID; and enhances E-cadherin levels, thereby reducing migration/adhesion of RAS transformed cells with EMT (48)

**Figure 2:**
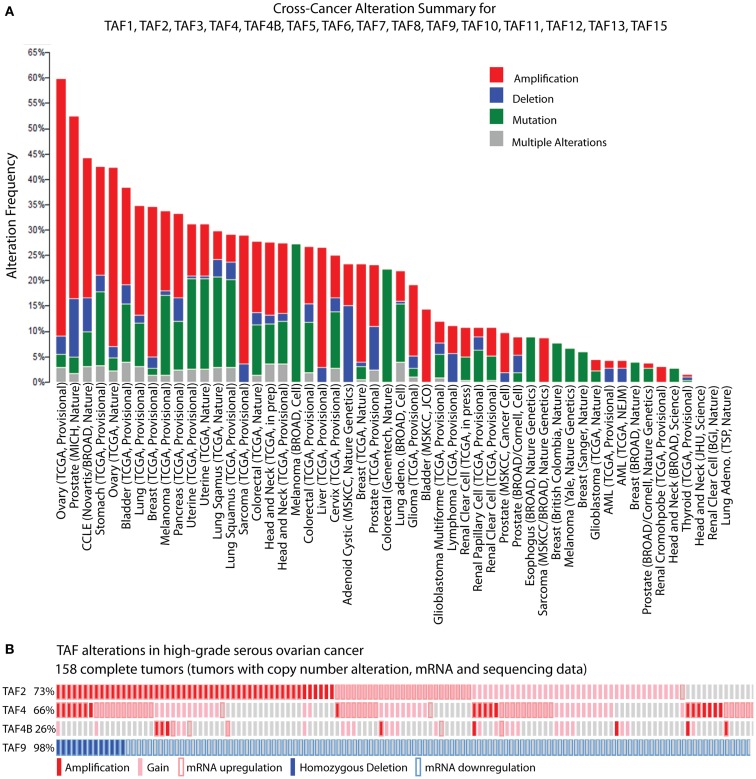
**TBP-associated factor alterations in high-grade serous ovarian cancer (HGSC)**. **(A)** Cross-cancer summary of copy number alterations and mutations in TAFs for all cancer sets in the cBioPortal for Cancer Genomics (2, 3). **(B)** Amplifications, copy number gains, and mRNA upregulation of TAF2, TAF4, and TAF4B, and deletions and mRNA downregulation of TAF9, in the cBioPortal HGSC set (TCGA, provisional, complete tumors) (2, 3). These “oncoprints” are partial views of alterations in 158 complete tumors. Alterations are present in 158 complete tumors in the percentages noted on the left.

While biochemical and genetic analyses point to a role for TFIID in regulating tumor-related phenotypes, the recent assembly of the cBioPortal for Cancer Genomics by MSKCC sheds further light on the subject. A bioinformatic analysis of 316 high-grade serous ovarian carcinomas (HGSC) whose copy number, mutational status, and mRNA levels are stored in the portal, reveals the TFIID complex as one of the most significantly altered subnetworks in this panel of tumors; TFIID alterations are detected in 42% of tumors by the study’s analyses (49). Interestingly, when comparing the incidence of TAF copy number alterations (CNAs) and mutations between various tumor types, the HGSC set [The Cancer Genome Atlas (TCGA); provisional; all tumors] exhibits the most frequent alterations (59.8%). The overwhelming majority of these alterations are amplifications (Figure 2A), supporting the argument that the TFIID complex is important in ovarian cancer. A closer look at specific TAF alterations in the HGSC set (TCGA; provisional; complete tumors = all tumors with CNAs, mRNA, and sequencing data) reveals TAF2 amplifications, copy number gains, and mRNA upregulation (*Z*-score >+2.0) in 73% of tumors; TAF4 and TAF4B show these alterations in 66 and 26% of tumors, respectively (2, 3) (Figure 2B). Other TAFs not depicted here are also frequently amplified. Conversely, homozygous deletions or mRNA downregulation (*Z*-score <−2.0) of TAF9 are observed in 98% of complete tumors (2, 3) (Figure 2B), which is in accordance with TAF9’s reported role as a cofactor for p53 and its involvement in promoting apoptosis (44, 45). As suggested by this TCGA data, as well as the studies outlined in Table 1, particular TAFs may regulate tumor properties in very precise and tissue-specific manners. Moreover, studies performed on the role of TAF subunits in development suggest that specific TAF subunits function as master regulators of differentiation and proliferation (11–13, 50–58), which has important implications for the process of dedifferentiation that occurs with tumor progression. Herein, we outline the potential for TAFs to regulate differentiation, proliferation, and apoptosis in ovarian tumors, and discuss the implications of this regulation for tumor cell-autonomous and microenvironment effects.

## Materials and Methods

### cBioPortal for cancer genomics

The portal assembled by the MSKCC at http://www.cbioportal.org/public-portal/ was used for analysis of TAF alterations in ovarian cancer. To generate the cross-cancer alteration summary, “Mutation and CNA” data types were analyzed for “All Cancer Studies.” TAF1, TAF2, TAF3, TAF4, TAF4B, TAF5, TAF6, TAF7, TAF8, TAF9, TAF10, TAF11, TAF12, TAF13, and TAF15 were input as the gene set to be analyzed. To examine alterations in individual TAFs, we queried “Ovarian Serous Cystadenocarcinoma” (TCGA, Provisional). One hundred fifty-eight “complete tumors” (those with copy number, mRNA expression, and sequencing data) were included. For TAF2, TAF4, and TAF4B, the following search was performed: DATATYPES: AMP GAIN EXP > 2.0; TAF2, TAF4, TAF4B, in order to view amplifications, copy number gains, and mRNA expression (*Z*-score > 2.0) for these TAFs. No mutations were present. Copy number data is putative and generated by GISTIC algorithm. Messenger RNA expression *Z*-scores were determined by RNA Seq Version 2 RSEM. Detailed information on the GISTIC algorithm and mRNA *Z*-scores can be found at http://www.cbioportal.org/public-portal/faq.jsp.

### Animals

All animal protocols were performed at Brown University. Mice were killed by carbon dioxide euthanasia, and all protocols were reviewed and approved by the Brown University Institutional Animal Care and Use Committee.

### Surgeries and liver collection

Mouse hepatectomies were performed as described in Greene and Puder (59). Briefly, adult mice were anesthetized using insoflurane prior to surgery. Fifty percent hepatectomy consisted of ligature and removal of the left and left medial lobe through a mid-abdominal incision followed by suture of incision. Sham surgeries involved incision and manipulation of liver, without removal, followed by suture of incision. Mice recovered in cages on a 37° warm plate during the hours following surgery. In the experimental mice, the left lobes were collected at 0 h (“removed”), and the right lobes were removed at the indicated timepoints (“recovered”). Total RNA was isolated for qRT-PCR analysis.

### Human granulosa cell tumors

Samples of human granulosa cell tumors (GCTs) were obtained from the Ottawa Ovarian Cancer Tissue Bank, with consent from the patients and under a protocol approved by The Ottawa Hospital Research Ethics Board (1999540-01H). Frozen tissues were homogenized in RLT buffer using an IKA Ultra-Turrax homogenizer and total RNA was extracted using the Qiagen RNeasy Mini Kit as per the manufacturer’s protocol.

### Quantitative RT-PCR

cDNA was prepared from 1 μg total RNA, and qRT-PCR performed as previously described (33). Data was analyzed using the ΔΔCt method and normalized to 18s rRNA. Correlation between *TAF4* or *TAF4B* and *CCND2* was determined by Pearson Correlation Co-efficient (R). *P*-values were determined by two-tailed, unpaired Student *t*-test. Primers used are as follows:

Mouse *Tafa4* F – ATC TCC ACT GTG CAG GCTT CC
Mouse *Taf4a* R – GGT CAG CTG CCG TGC AAT A
Mouse *Taf4b* F – GAT GTT ACT AAA GGC AGC CAA GAG T
Mouse *Taf4b* R – CTG CTC TGG ATC TTC TTT ATT GGA G
Human *TAF4* F – CTC AGA ACC CGA CCA ACA TCC
Human *TAF4* R – CTT CGG ACG AGG ACC ATT CC
Human *TAF4B* F – ATC CAG TTT CCT GCT AAT TTG C
Human *TAF4B* R – CCA ACA TCA ACG GAC CAC TGT
Human *CCND2* F – CCG ACA ACT CCA TCA AGC CT
Human *CCND2* R – AGCACCACCAGTTCCCACTC
18s rRNA F – CCG CGG TTC TAT TTT GTT GG
18s rRNA R – GGC GCT CCC TCT TAA TCA TG

### Antibodies and western blot analysis

Mouse ESC protein extracts pre- and post-retinoic acid (RA)-induced differentiation were generously provided by the Fairbrother lab (Brown University) and prepared as described in Tantin et al. (60). Western blot analysis was performed as previously described (33). Antibodies used are as follows: (1) TAF II p250 (1:200; sc-17134; Santa Cruz); (2) mouse monoclonal anti-TAF(ii)135 (1:250; 612054; BD Transduction Laboratories); (3) polyclonal rabbit anti-mouse TAF4B [1:250; raised against amino acids 1–98 (N-terminus/co-activator domain) of mouse TAF4B (33, 61, 62)]; (4) TBP (ab818; Abcam); (5) rabbit anti β-tubulin (1:200; RB-9249-P; Thermo Scientific).

## TFIID as a Direct Regulator of Cellular Differentiation States

**Figure 3:**
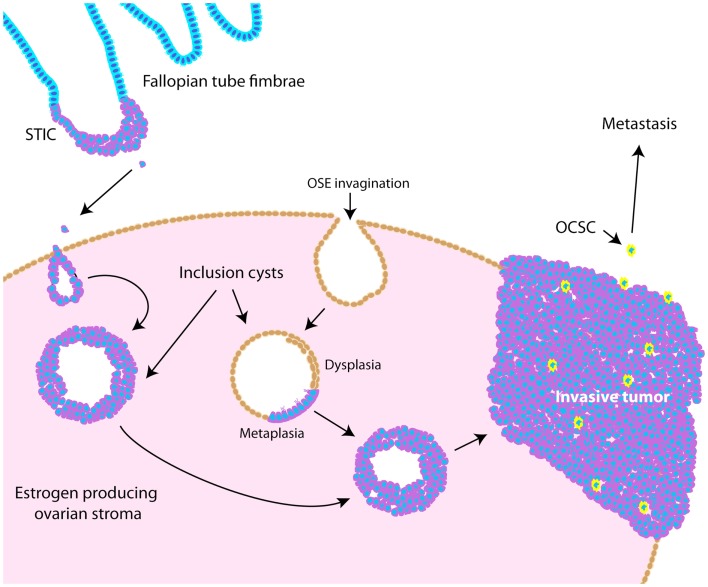
**Schematic illustrating pathogenesis and dedifferentiation in epithelial ovarian cancer (EOC)**. Epithelia on the fallopian tube fimbrae may become transformed, causing the development of a serous tubal intraepithelial carcinoma (STIC). These cells may eventually slough off the fimbrae and become lodged within the ovarian stroma, causing an inclusion cyst lined with epithelia resembling that of the fallopian tube. Alternatively, invaginations of the OSE can cause inclusion cysts, which become dysplastic and then undergo metaplasia to resemble fallopian tube epithelium. These cysts can eventually develop into tumors that undergo dedifferentiation as they progress. Ovarian cancer stem-like cells are thought to represent a small proportion of the tumor bulk (illustrated as yellow cells), and are more likely to metastasize.

Throughout ovarian tumorigenesis and progression, tumor cells undergo multiple requisite changes in morphology and phenotype (Figure 3). The ovarian surface epithelium (OSE) undergoes metaplasia to a fallopian tube epithelial morphology early in serous adenocarcinoma formation (63) and is associated with an increase in E-cadherin expression as the mesothelial OSE obtains the columnar epithelial phenotype of the fimbrial epithelium (63). The process of metaplasia is controversial as evidence has accumulated that many HGSC develop from fimbrial epithelial cells that become lodged in the ovarian stroma (64). However, convincing arguments still exist for OSE metaplasia, including the fact that the process can be replicated in mouse and hen models of ovarian cancer (63, 65–70). The initial origin of the tumor becomes less important as it progresses from a well-differentiated papillary histology to a dedifferentiated morphology. This process of dedifferentiation from an organized morphology resembling the tissue of origin to a disorganized mass of less differentiated cells is a hallmark of cancer progression in diverse tissues (71–74), and is defined by a grading system set forth by the International Federation of Gynecology and Obstetrics (FIGO) (75). Poorly differentiated tumors are associated with a worse prognosis (76–79) since dedifferentiation allows adaptive changes in morphology that promote invasion and metastasis (71). Thus, tumor dedifferentiation is closely associated with the process of epithelial-to-mesenchymal transition (EMT), whereby loss of epithelial differentiation allows cells to acquire productive characteristics, such as invasion and migratory capabilities (80). In ovarian cancer, dedifferentiated tumors manifest as a solid tumor mass, lacking the glandular morphology seen in well-differentiated serous ovarian tumors (81). Accordingly with loss of epithelial morphology, E-cadherin expression is frequently reduced in dedifferentiated tumors, ascites, and metastases (72, 82–84). In dedifferentiated tumors where E-cadherin is not lost, other adhesion complex components, such as the catenins, could be disrupted (85, 86). Collectively, these studies show that ovarian tumors undergo complex morphological and functional changes from their initiation to progression.

Ovarian cancer stem-like cells (OCSCs) represent a very small percentage of tumor cells and are considered a dedifferentiated, or pluripotent, cell-type. Accordingly, CSCs are characterized by upregulation of mRNAs encoding stem cell markers, including *OCT4, SOX2*, stem cell factor receptor (*C-KIT*), *NOTCH1, ABCG2, BMI1*, and *NES* (Nestin) (87) and NANOG protein levels (88). Functionally, CSCs can recapitulate the original tumor, form xenografted tumors at limiting dilutions, are resistant to chemotherapy, form spheroids in culture, and have increased invasive and migratory potential (89). OCSCs have been shown to be enriched for CD44^+^/CD117 (C-KIT)^+^ cells (87, 90), CD44^+^/CD24^−^ cells (91), and CD44^+^/MyD88^+^ cells (90). More recently, ALDH1 and CD133 have been identified as markers for OCSCs (92–94). Nonetheless, it is challenging to study this population of cells since it represents such a small percentage of the tumor mass. Since dedifferentiated tumors are further along the path toward pluripotency, perhaps CSCs represent a small proportion of cells that are furthest along that path. In support of this notion, Gabbert et al. noted that the “invasion front” of colon carcinomas was marked by a loss of differentiation, and that in already undifferentiated colon carcinomas, only subtle changes were required for the invasive phenotype (71). While it is clear that tumors undergo these various dedifferentiation processes throughout tumorigenesis and progression, the precise molecular events regulating metaplasia, dedifferentiation, EMT, and the establishment of OCSCs are not well-understood. Understanding these mechanisms is essential if we hope to identify treatments that inhibit or reverse these processes.

**Figure 4:**
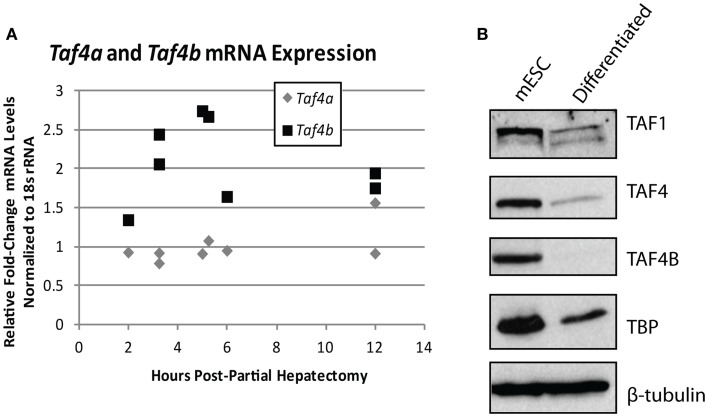
**TBP-associated factor subunits are dynamic during differentiation and proliferation**. **(A)** Quantitative RT-PCR analysis of *Taf4a* and *Taf4b* in normal (removed) and partial hepatectomy (recovered) mouse livers. Data represented as fold-increase (recovered/removed) plotted against hours post-hepatectomy. **(B)** Western blot analysis of TAF1, TAF4, TAF4B, and TBP in mouse embryonic stem cells pre- and post-retinoic acid-induced differentiation. β-tubulin was used as a loading control.

The importance of TAFs in regulation of normal differentiation processes during development has been well-established in recent years. One of the first studies to indicate the involvement of TAFs in differentiation shows a reduction in TAF4 expression in embryonic cortical neuronal stem cells that differentiate down a neuronal, but not a glial differentiation pathway (50), suggesting that specific TAFs are involved in particular differentiation pathways. Comparatively, TAF10 is essential for the differentiation of keratinocytes, but is dispensable in adult epidermis (51), and is also required for proliferation of undifferentiated embryonic carcinoma cells, but not after *in vitro* differentiation of these cells by RA (95). These two studies establish the importance of specific TAFs in regulating proliferation and differentiation of progenitor, but not differentiated cell-types. In parallel, knockdown of TAF1 and TAF4B in primary mouse embryonic maxillary mesenchymal cells reduces proliferation and causes deregulation of osteogenic differentiation (52). In liver, lower levels of TBP and TAFs are seen in hepatocytes compared to differentiated hepatoblasts and TAF4 and TBP are reduced upon *in vitro* induced hepatogenesis (53). These results are in agreement with the typically low levels of TAF4B detected in adult liver that we have previously reported (17). Accordingly, we saw an increase of *Taf4b*, but not *Taf4a* (the mouse homolog of human *TAF4*), mRNA upon liver regeneration induced by partial hepatectomy, suggesting that this specific TAF subunit may be involved in regulating either hepatocyte re-entry into the cell cycle or differentiation of liver stem cells (Figure 4A). Collectively, these studies generally reveal a reduction of TAFs as cells progress from a pluripotent to a differentiated state, suggesting their importance in regulating this process or in maintenance of pluripotency.

TBP-associated factors can act outside of the canonical TFIID complex to regulate differentiation as well. Deato et al. show that an alternate TFIID complex consisting of TAF3 and TBP-related factor 3 (TRF3) is required for activator-dependent transcription of myogenin and differentiation of myoblasts to myotubes. Importantly, expression of other TFIID subunits typically believed to be essential for TFIID function, including TAF1, TAF4, and TBP, is lost with myoblast differentiation (11–13). Moreover, higher levels of TAF3 are reported in mouse embryonic stem cells (mESCs) compared to mouse embryonic fibroblasts (MEFs), adult spleen, lung, heart, brain, and liver. In a study by Liu et al., TAF3, along with the pluripotency factor OCT4, are reduced upon mESC differentiation, accompanied by slight reductions in TAF4 and TBP. Confirming the importance of TAF3 in mESC differentiation, when TAF3 is knocked down in plated embryoid bodies, the cells lose pluripotency, and largely differentiate to neurons. Interestingly, the authors identify a TFIID-independent transcriptional regulatory role for TAF3, which works in conjunction with CCCTC-binding factor (CTCF) and cohesin to regulate transcription of endoderm-specific gene expression programs (54).

The TAF4 and TAF4B subunits of TFIID are also known to support pluripotency. Bahat et al. recently reported that the TAF4 paralog TAF4B is highly expressed in mESCs and is downregulated upon differentiation (55). We have independently observed the reduction of TAF1, TAF4, TAF4B, and TBP upon *in vitro* differentiation of mouse embryonic stem cells by RA (Figure 4B). Moreover, the Bahat et al. study reveals that TAF4B knockdown decreases mESC self-renewal, differentiation, and cell cycle progression, along with a reduction in genes that are also reduced upon RA-induced mESC differentiation. Conversely, they report TAF4 knockdown to increase proliferation and prevent RA-induced differentiation (55), suggesting functional differences between these paralogs in mESCs. Mengus et al. report an increase in serum-independent autocrine growth upon TAF4 knockdown in MEFs, which they attribute to compensation by TAF4B (56), suggesting some functional overlap between these paralogs. However, like Bahat et al. they also show that there are differences between TAF4 and TAF4B in MEFs, as illustrated by deregulation of over 1,000 genes in the TAF4 knockdown cells that are not compensated for by TAF4B (56). Although these studies approach TAF knockdowns from opposite ends of the differentiation spectrum, they both reveal interesting information about the differences and similarities between TAF4 and TAF4B, and point to a role for these TAFs in regulating the balance of differentiation and/or proliferation.

Some of the results of the Bahat et al. study regarding TAF4 are in contrast to a related study by Pinjappel et al., in which the authors show that knockdown of TAF1, TAF3, TAF4, TAF4B, TAF5, TAF6, TAF9, TAF11, TAF12, and TAF13 each results in differentiation of mESCs, but does not affect proliferation or apoptosis (57). TAF5 knockdown alone decreases transcription of pluripotency genes, increases transcription of differentiation markers, and decreases OCT4/NANOG binding at the TAF4 promoter, pointing to transcriptional activation of TAF4 as essential in maintaining stemness. Overexpression of TAFs during OCT4/SOX2/KLF4/MYC (OSKM) reprograming of MEFs increases mESC morphology, while omission of TAF4 from the overexpressed complex abolishes its stimulatory effect on reprograming. Eventually, it was found that TAF4 alone promotes the reprograming of MEFs to induced pluripotent stem cells (iPSCs), highlighting the importance of this individual TAF in promoting cellular reprograming to a dedifferentiated state (57). However, the main discrepancy between these studies – TAF4 knockdown increasing proliferation and preventing differentiation of mESC versus TAF4 knockdown not affecting proliferation yet promoting differentiation of mESC – needs to be addressed. Interestingly, alternative TAF4 isoforms could play a role in modulating differential functions of TAF4. Kazantseva et al. show that expression of TAF4 isoforms with structural modifications of the TAF4-TAFH (co-activator) domain increases during differentiation of human mesenchymal stem cells, while silencing of TAF4-TAFH intact isoforms causes cell cycle arrest and blocks specific differentiation pathways (58). Moreover, the expression of the TAF4-TAFH deletion isoforms is also cell-type specific, with one of them (TAF4_v4) being expressed only in the ovary, placenta, stomach, testis, and thymus (58). This study highlights another layer of complexity to transcriptional regulation by TAF subunits that is introduced by alternative splicing events, and also suggests the importance of taking a tissue-specific approach to studying this regulation.

**Figure 5:**
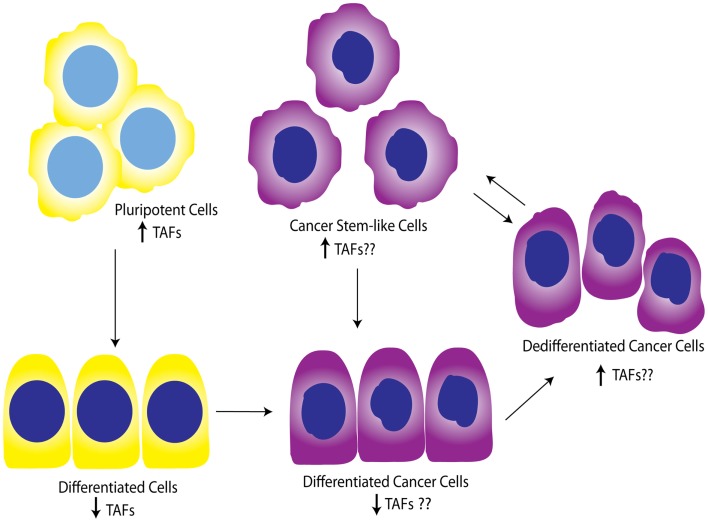
**Working model for TAF involvement in differentiation during development and tumor progression**. Studies show that TAFs are downregulated in differentiated cells compared to pluripotent cells. This model illustrates the hypothesis that TAFs are also expressed at lower levels in differentiated cancer cells, but may be upregulated as these cells progress to dedifferentiated cells or pluripotent cancer stem-like cells. Yellow, normal cells; purple, cancer cells.

While the contribution of TAFs to ovarian proliferation and differentiation has not been well-investigated, these studies support the general notion that certain core TAFs are reduced upon differentiation. It is not clear whether other differentiation signals cause the downregulation of TAFs, or the downregulation of TAFs promotes differentiation signals; however, a productive feed-forward loop likely exists between TAF subunits and OSKM factors, as suggested by Pijnappel et al. Regardless, it is apparent that TAFs play an important role in maintenance of pluripotency and cause dedifferentiation when overexpressed in differentiated cells. It is also clear that diverse TAFs function to regulate differentiation in tissue-specific manners, highlighting the importance of expanding these studies to include the ovary, and potentially other tissues. In relationship to cancer, TAF regulation of differentiation state could be important as tumors dedifferentiate as a whole, or as individual cells take on a more “pluripotent” phenotype and become CSCs. Figure 5 illustrates a potential model for TAF regulation of differentiation and proliferation as it pertains to development and ovarian tumor progression. As outlined here, we hypothesize that deregulation of TAF expression, such as what occurs with amplifications commonly seen in HGSC, contributes to tumor dedifferentiation or establishment of OCSCs.

## TAF Regulation of Tumor-Related Phenotypes

The contribution of individual TAFs to tumor dedifferentiation as well as diverse tumor properties and their microenvironment needs to be considered. For the sake of this discussion, we will focus on TAF2, TAF4, TAF4B, and TAF9 as illustrative examples of how specific TAF subunits may be involved in regulating ovarian tumor properties. TAF2 appears to be important in ovarian cancer since it is most frequently altered in HGSC, with amplifications, copy number gains, and mRNA upregulation present in 73% of tumors (2, 3). Although little is known about the role TAF2 might play in cancer, it is required for cell cycle progression in yeast, and expression of yeast TAF2 and several other TAFs is dramatically reduced when cells are induced into a stationary phase (31). From a biochemical standpoint, the TAF1/TAF2 complex recognizes Initiator (Inr) elements (96), the relevance of which becomes apparent when examining another study showing that TATA-binding defective TFIID can still initiate transcription from TATA-containing or TATA-less promoters that have an Inr sequence (97). Although this study utilized a mutant TBP that was still capable of binding TFIID (97), a study by Wright et al. investigating the stability of the TFIID complex in *Drosophila* shows that TBP knockdown does not affect the stability of the TFIID complex, suggesting that TBP is not strictly required for TAF assembly (98). Figure 1A illustrates a TBP-free TFIID sub-complex (TFTC) (99) that could be responsible for TAF1/TAF2 regulation of transcription from Inr consensus sequences. Importantly, the set of genes containing an Inr sequence overlaps with, yet is distinct from, those bound only by non-defective TFIID (97), suggesting that a distinct transcriptional profile could exist in TAF2-overexpressing cells. In support of this notion, Dehm et al. demonstrate that the TAF1/TAF2 complex binds *C-SRC* 5′ exon promoters, which are TATA-less and contain Inr sequences (27). Interestingly, *C-SRC* overexpression and activation are reported in ovarian cancer (100, 101) and contribute to activation of growth factor signaling cascades (102, 103) and anti-estrogen resistance (100). Given these results, it is possible that TAF2 overexpression could increase transcription of *C-SRC* in some ovarian tumors.

Reminiscent of the TFIID-independent function of TAF3 in regulation of myogenesis (11–13), the transcriptional control of Inr-containing promoters by TAF1/TAF2 may be relevant in ovarian cancer, since TBP is predominantly downregulated in many HGSCs (2, 3). Incidentally, reliance on TBP-independent TAF functions is one potential reason for this unexpected downregulation of TBP; another could actually be mutation of p53 in HGSC, since TBP is known to bind and derepress p53 (104). Interestingly, TAF1 is not commonly deregulated in HGSC, although its role as a tyrosine kinase and regulator of cell cycle and apoptosis in other tissues is well-described (19–21, 23–25, 30, 105–107), and it is also frequently mutated in uterine serous carcinoma (18). The apparent tissue-specific effects of TAF1 reiterate the importance of studying individual TAFs in tissue-specific contexts. Further dissection of the role TAF2 plays in ovarian cancer could illuminate whether this TAF regulates alternative transcriptional programs involved in differentiation and proliferation.

After TAF2, TAF4 is the most frequently amplified and overexpressed TAF in HGSC (2, 3). We have already discussed its role in driving MEF dedifferentiation to iPSC (57), suggesting it could be a major regulator of dedifferentiation/pluripotency in certain contexts. From a molecular standpoint, TAF4 is interesting for several other reasons as well. In *Drosophila*, it is the most crucial subunit for maintaining the stability of the holo-TFIID complex, with TAF5, TAF6, TAF9, and TAF12 also contributing to this core complex. RNAi-mediated knockdown of TAF4 or TAF12, which dimerize via their histone-fold domains (HFDs), results in degradation of most of the other TAF subunits (98). Interestingly, TAF1, TAF2, and TAF11 are less critical (98), which is in support of the idea that a TAF1/TAF2 complex may initiate transcription as part of a TFIID sub-complex at Inr elements. Likewise, TAF1 and TAF4 are important for directing transcription from TATA-less, downstream promoter element (DPE)-containing promoters (98) (Figure 1B), while TATA-containing promoters are less dependent on these subunits. This evidence suggests that disruption of TAF4’s HFD interaction with the HFD of TAF12 could potentially destabilize both holo-TFIID and many TFIID sub-complexes. The overexpression of many TAFs in HGSC discussed previously raises the possibility that inhibition of TFIID-driven transcription, such as might be possible using small molecule inhibitors, could reduce expression of a large set of genes potentially important for ovarian tumor growth and differentiation. However, compensation by alternative TFIID complexes might instead drive some level of transcription under these circumstances, including a complex containing the TAF4 paralog TAF4B, which is depicted in Figure 1C.

**Figure 6:**
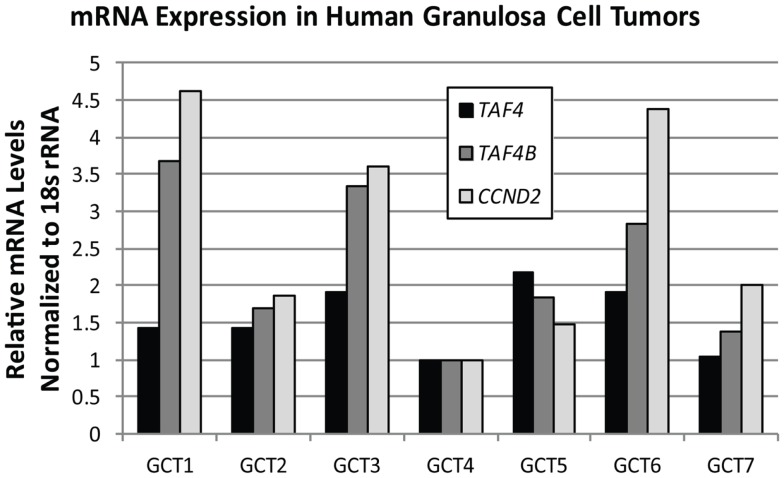
***TAF4B* correlates with *CCND2* mRNA levels**. Quantitative RT-PCR analysis of *TAF4A* (black bars), *TAF4B* (dark gray bars), and *CCND2* (light gray bars) mRNA levels in human granulosa cell tumors. Pearson correlation co-efficient (*R*) for *TAF4B* and *CCND2* = 0.93; *TAF4A* and *CCND*2 = 0.32.

TAF4B is not as frequently altered in ovarian cancer, but nonetheless exhibits amplifications, copy number gains, or mRNA upregulation in 25% of HGSC (2, 3). TAF4B is relevant to the discussion of TAFs in ovarian cancer because its expression is enriched in the ovary compared to other tissues, and it is required for proper ovarian follicle development and murine fertility (17, 61, 62, 108). In addition to oocyte and folliculogenesis defects in the *Taf4b*-null ovary, granulosa cell proliferation and survival are dependent upon TAF4B expression (62). It is still not clear, however, whether adult *Taf4b*-deficient ovarian defects are established during early development or occur due to signals from defective oocytes that remain into early adulthood in the *Taf4b*-deficient ovary. Likewise, the precise contributions of germ cell versus somatic factors are not well-understood. Studies are underway to elucidate the mechanisms underlying the *Taf4b*-null infertile phenotype. It is clear, however, that TAF4B plays a role in regulating proliferation in some contexts. The gene for Cyclin D2, a key cyclin that selectively regulates both normal granulosa cell and GCT proliferation (109), was identified as a transcriptional target of TAF4B by chromatin immunoprecipitation (ChIP) of TAF4B at the *Ccnd2* promoter in TAF4B-overexpressing rat spontaneously immortalized granulosa cells (SIGCs) (110). In human GCTs, *TAF4B* mRNA expression strongly correlates (*R* = 0.93) with expression of *CCND2*, while TAF4 was found to only weakly correlate to *CCND2* levels (*R* = 0.32; Figure 6). These data suggest that TAF4B regulation of this granulosa cell cyclin could play a role in GCT proliferation. Other genes linked to ovarian cancer were also identified as TAF4B targets in TAF4B-overexpressing SIGCs, including *c-Jun*, matrix metalloproteinase-3 (*Mmp-3*), and Fibronectin-1 (*Fn1*) (110). The preferential regulation of these genes by TAF4B over TAF4 could be due to a slight alteration in conformation of TAF4B-containing TFIID complexes compared to TAF4 alone containing complexes, which can affect promoter occupancy of specific genes, as identified by Liu et al. for c-Jun (111).

In further support of a potential role for TAF4B in GCT, we have recently found that TAF4B is upregulated by estrogen in normal mouse granulosa cells (33). Since estrogen signaling could contribute to GCT pathogenesis (112), perhaps TAF4B is an effector of certain aspects of estrogen signaling in GCT. Execution of estrogen-dependent effects by TAFs is not without precedence, as TAF10 was found to be important for efficient transcription at ERE-containing promoters in MCF7 breast cancer cells (47). From these data showing TAF4B involvement in estrogen signaling, granulosa cell proliferation, and regulation of a cell cycle protein, it seems likely that it could play a role in GCT; since granulosa cells also support epithelial ovarian cancers (EOC) as the microenvironment in which inclusion cysts arise, we will also discuss its potential involvement in EOC pathogenesis.

One long standing hypothesis for how the ovarian milieu contributes to ovarian cancer growth is hormonal signaling within the ovary, especially estrogen signaling. The granulosa cells are the estrogen producing cells in the ovary, and could support tumorigenesis from inclusion cysts, whether the cysts arise from the OSE or fimbrial epithelium. Studies report the stimulatory effects of estrogen on OSE hyperplasia (113) and metaplasia (114) as well as the more rapid establishment of serous ovarian tumors by SV40 Large T-antigen driven transformation of the OSE upon exposure to exogenous estrogen (66). In support of these experimental studies, epidemiological studies show that women who have been on long-term estrogen hormone replacement therapy have a greater risk of developing ovarian cancer (115–120). Our recent study finding TAF4B to be upregulated by estrogen in granulosa cells also reports dramatic upregulation of TAF4B in estrogen-supplemented mouse tumors (33). It is not clear, however, whether the upregulation of TAF4B in these tumors occurs in the OSE-derived cells (suggesting cell-autonomous effects) or in the tumor stroma (suggesting microenvironment effects). Either of these scenarios is possible, since we see TAF4B expression in both OSE and fimbrial epithelium in addition to the granulosa cells. Moreover, while TAF4B is normally regulated by ERβ in granulosa cells, it can also be regulated by ERα in the absence of ERβ (33). Collectively, these results do not exclude either a cell-autonomous or microenvironment role for TAF4B in EOC.

Another scenario exists whereby TAF4B could regulate the ovarian microenvironment to promote tumorigenesis. TAF4B was originally identified as a TFIID subunit in B-cells (14) and promotes expression of anti-apoptotic genes by acting as a co-activator for NFκB. In this manner, TAF4B and NFκB work cooperatively to suppress tumor necrosis factor-α (TNFα)-mediated apoptosis in 293 cells *in vitro* and in B- and T-cells in mice (121, 122). While this mechanism has not been investigated in the ovary, it could represent another way that TAF4B contributes to regulation of the immune microenvironment and protection from apoptosis, particularly in ovarian tumors with constitutively activated NFκB signaling. Such constitutive activation occurs in ovarian cancer due to the chronically inflamed microenvironment (123), resulting in enhanced growth and protection from apoptosis (112). Interestingly, NFκB activation may also be a hallmark of OCSCs and contribute to their escape from apoptosis. Alvero et al. show that CD44^+^/MyD88^+^ cells have constitutive activation of NFκB, and when treated with paclitaxel or TNFα, upregulate NFκB signaling instead of undergoing apoptosis (90). It will be interesting to determine if TAF4B also contributes to this NFκB-mediated protection of OCSCs.

While TAF4B could potentially be involved in mediating anti-apoptotic effects, TAF9 is illustrative of a TAF acting as a putative tumor suppressor, since it is downregulated or deleted in 98% of HGSC. In support of this role, TAF9 is known to be a co-activator for p53 (44–46), which is ubiquitously mutated and downregulated in HGSC (2, 3). TAF9 physically interacts with p53 at its N-terminus, where p53 also interacts with its negative regulator, MDM2, thereby inhibiting MDM2 degradation of p53. Functionally, this interaction translates to an increase in p53-induced cell cycle arrest or apoptosis, as demonstrated by fibroblast growth arrest following TAF9 overexpression, UV-induced association of p53 with TAF9 (45), and TAF9-induced apoptosis of neuroendocrine tumor cells deprived of nerve growth factor (124). The pro-apoptotic role of TAF9 is illustrative of the complexity of regulation by TAFs and the multitude of effects that are likely species-, cell-type-, and context-dependent; i.e., while knockdown of TAF9 affects differentiation of mESCs (57), TAF9 can also regulate apoptosis in different contexts (44, 45). It will be important to determine if the downregulation of TAF9 in HGSC is a consequence of *TP53* mutations, or if alterations in TAF9 are observed in other subtypes of EOC, which lack the hallmark p53 mutation of HGSC (125, 126).

As discussed in this section, individual TAFs have the potential to regulate tumor properties by a variety of mechanisms. This may include regulation of differentiation, as discussed in the previous section, or could also involve cell cycle effects, apoptosis or metastasis. The contribution of TFIID stability, presumably regulated by histone-fold dependent association of TAF4 and TAF12, as well as the contribution of putative TFIID sub-complexes that lack TBP or other subunits, merit investigation in the context of ovarian cancer. These complexes could have important roles in translating signals from upstream oncogenic cascades that are deregulated in ovarian cancer, including those regulating proliferation and differentiation.

## Conclusion

Herein, we have outlined a hypothesis that TAF subunits, because of their documented importance in dedifferentiated cell-types and loss of their expression occurring with differentiation, also play a functional role in driving tumor dedifferentiation. TFIID, and TAF2, TAF4, TAF4B, and TAF9 in particular, are under-explored as potential contributors to the dedifferentiation process, and could also contribute to regulation of proliferation and apoptosis. Measuring levels of TAF subunits throughout tumor initiation and progression could reveal if they are modulated according to the differentiation state of the tumor. In addition to testing the role of TAFs in the differentiation of ovarian cell-types, differences between GCT versus EOC and cell-autonomous effects versus microenvironment effects should be explored.

It is crucial to investigate new areas in ovarian cancer, especially avenues that target processes downstream of oncogenic signaling cascades. Indeed, in yeast, approximately 84% of genes require one or more TAFs for their expression (127), supporting the notion that tumor characteristics could heavily rely on proper TAF functioning. Perhaps disruption of this function could be used as part of combinatorial therapy in ovarian cancer. However, research will also need to be done to investigate adaptive transcriptional mechanisms that could circumvent the reduction of TAF activity. Furthermore, the TAFs associated with RNA polymerase I and III TBP-containing general transcription complexes could also be relevant to the study of ovarian cancer (128, 129), since these other polymerases directly promote cellular growth (130). We conclude that a greater understanding of mechanisms of transcriptional regulation that carry out signals from oncogenic signaling cascades is needed in order to expand our understanding of the etiology and progression of ovarian cancer, and uncover new methods of treatment for this disease.

## Conflict of Interest Statement

The authors declare that the research was conducted in the absence of any commercial or financial relationships that could be construed as a potential conflict of interest.
